# Gut microbiota and the gut–liver axis in MASLD: mechanisms, immune regulation and therapeutic opportunities

**DOI:** 10.3389/fphar.2026.1917467

**Published:** 2026-08-13

**Authors:** Jingjing Liu, Mei Zhao, Shujing Wang, Chengyong Zhang, Yingli Zhong, Yuanjun Qu

**Affiliations:** Department of Pharmacy, Affiliated Hospital of Shandong University of Traditional Chinese Medicine, Jinan, China

**Keywords:** gut microbiota, immune regulation, medicine, metabolic dysfunction-associated steatotic liver disease, microbial metabolites

## Abstract

Metabolic dysfunction-associated steatotic liver disease (MASLD) is one of the most common chronic liver diseases worldwide, affecting more than one-third of adults and imposing a substantial hepatic and systemic metabolic burden. With the continuing rise in obesity, type 2 diabetes mellitus, and related metabolic abnormalities, the prevalence of MASLD is expected to keep increasing. This review summarizes MASLD from the overarching perspective of gut-liver axis dysregulation, focusing on alterations in gut microbial composition and function, intestinal barrier injury, disturbances in microbiota-derived metabolites, and gut microbiota-associated epigenetic and immune dysregulation during disease initiation and progression. It further integrates the overall significance of these changes in the evolution of MASLD. Finally, potential therapeutic strategies represented by fecal microbiota transplantation and natural products are briefly discussed, suggesting that targeting the gut microbiota and its related networks may provide new opportunities for MASLD prevention and treatment.

## 1 Introduction

Metabolic dysfunction-associated steatotic liver disease (MASLD) is a class of disorders characterized by hepatic steatosis in the presence of metabolic dysfunction (European Association for the Study of the Liver et al., 2024). Given that the 2023 multi-society Delphi consensus recommended replacing nonalcoholic fatty liver disease (NAFLD) and nonalcoholic steatohepatitis (NASH) with MASLD and metabolic dysfunction-associated steatohepatitis (MASH), respectively (Rinella et al., 2023), MASLD-related terminology is used throughout this review. However, when the cited literature retains the terms NAFLD, NASH, or metabolic dysfunction-associated fatty liver disease (MAFLD), the original terminology is preserved. Meta-analysis of studies using the former NAFLD definition estimated a global prevalence of 30.05% between 1990 and 2019, reaching 38.00% in studies conducted during 2016–2019 (Younossi et al., 2023). The associated economic burden is also substantial, with annual direct medical costs estimated at approximately US$103 billion in the United States and €35 billion across Germany, France, Italy, and the United Kingdom (Younossi et al., 2016). The pathogenesis of MASLD is closely associated with alterations in lipid metabolism and increased inflammation (Wang et al., 2024). Disease progression is continuous, with simple steatosis potentially evolving into the more severe state of MASH and, if left uncontrolled, further advancing to liver fibrosis, cirrhosis, and even hepatocellular carcinoma (Lekakis and Papatheodoridis, 2024).

Accumulating evidence indicates that inflammatory amplification in MASLD is not confined to the liver but is closely linked to dysregulation of the gut-liver axis (Hsu and Schnabl, 2023). The gut-liver axis refers to a bidirectional interactive system between the intestine and the liver that is established through anatomical, immune, metabolic, and microbial pathways (Hsu and Schnabl, 2023). Unhealthy dietary patterns can promote gut microbial dysbiosis and impair the intestinal barrier, thereby increasing intestinal permeability and allowing microbe-related molecules and metabolites to enter the liver through the portal vein, where they induce inflammatory responses and exacerbate lipid accumulation and liver injury (Long C. et al., 2024; Castelnuovo et al., 2024). In addition, gut microbiota-derived metabolites can participate in the pathogenesis of MASLD by modulating host metabolism and signaling pathways (Long Q. et al., 2024). Recent studies have further shown that the gut microbiota and its metabolites can reshape hepatic transcriptional programs through epigenetic mechanisms, including DNA methylation, histone modifications, and non-coding RNA-mediated regulation (Ha et al., 2024). On this basis, microbiota-related signals may further influence bile acid receptor signaling, choline metabolism, and insulin sensitivity, while also driving imbalances in innate and adaptive immunity, thereby contributing to the initiation and progression of MASLD (Wei et al., 2024; Luo et al., 2025; Popov et al., 2024).

This review integrates current evidence on gut–liver axis dysfunction in MASLD and considers its mechanistic and translational implications for disease progression and therapeutic intervention.

## 2 Gut microbial dysbiosis and barrier dysfunction in MASLD

Microorganisms, including bacteria, viruses, fungi, archaea, and protozoa, colonize multiple sites throughout the human body and play important roles in immune regulation, inflammatory responses, and intestinal permeability. The gut microbiota is primarily composed of bacteria from the phyla Firmicutes (79.4%), Bacteroidetes (16.9%), Actinobacteria (2.5%), and Proteobacteria (1%) (Popov et al., 2024; Rinninella et al., 2019). The gut microecology is an important factor in the development of MASLD, and its pathological progression is closely associated with gut microbial dysbiosis (Leung et al., 2016). In patients with MASLD, increased abundance of *Bacteroides* and reduced levels of *Prevotella* are both closely associated with hepatic inflammation (Boursier et al., 2016). Gut microbial metabolites include bile acids (BAs) and short-chain fatty acids (SCFAs), among others. The gut microbiota can also promote the development of the host immune system by producing metabolites and microbe-associated molecular patterns (MAMPs). Studies have shown that gut microbial metabolites play an important role in the pathogenesis of MASLD (Chu et al., 2019). Therefore, elucidating the mechanisms through which beneficial and harmful microbial communities influence fatty liver disease is of particular importance.

### 2.1 Microbial dysbiosis and functional remodeling

A growing body of evidence indicates that the gut microbiota plays an important role in the onset and progression of MASLD. In mouse models susceptible to high-fat diet-induced disease, transplantation of microbiota from these animals into germ-free mice was sufficient to induce experimental MAFLD (Le Roy et al., 2013). In addition, gut-directed antibiotic intervention (Bergheim et al., 2008) and probiotic supplementation (Ritze et al., 2014) have been associated with reduced lipogenesis and attenuation of hepatic steatosis. However, the translational value of antibiotic-based microbiota modulation remains uncertain. A recent narrative review of SIBO treatment reported an overall eradication rate of 63% with rifaximin; however, most of the included studies lacked placebo or untreated controls, and the four small placebo-controlled trials did not demonstrate superiority over placebo (Maslennikov et al., 2026). In patients with biopsy-proven NASH, 6 weeks of rifaximin did not improve alanine aminotransferase levels, hepatic lipid content, or insulin sensitivity (Cobbold et al., 2018). Similarly, vancomycin reduced bacterial diversity and altered microbial pathways involved in short-chain fatty acid and bile acid metabolism in obese, prediabetic men, but did not improve insulin sensitivity or other major metabolic outcomes (Reijnders et al., 2016). Moreover, antibiotic treatment can lead to persistent expansion of antibiotic-specific resistance genes in the human gut microbiota, limiting the suitability of prolonged empirical antibiotic use for a chronic metabolic disease such as MASLD (Kang et al., 2021). More broadly, microbiome-directed interventions in humans have not consistently improved liver-specific endpoints: allogeneic FMT in 21 patients with NAFLD did not improve HOMA-IR or MRI-PDFF, although abnormal small intestinal permeability decreased in participants with elevated baseline permeability, and a 1-year synbiotic RCT altered fecal microbiomes without reducing liver fat or fibrosis markers (Craven et al., 2020; Scorletti et al., 2020). Patients with MAFLD and MASH commonly exhibit alterations in the gut microbiota, characterized by small intestinal bacterial overgrowth (Miele et al., 2009), reduced bacterial diversity (Astbury et al., 2020), and enrichment of Enterobacteriaceae and *Streptococcus*, together with alterations involving 11 of 47 taxa within the Bacteroidetes phylum (Ponziani et al., 2019). Therefore, rather than emphasizing isolated increases or decreases in individual taxa, this section focuses on reproducible functional alterations of the gut microbiota, because the specific microbial taxa and metabolites associated with NAFLD differ across human cohorts (Zeng et al., 2024).

**FIGURE 1:**
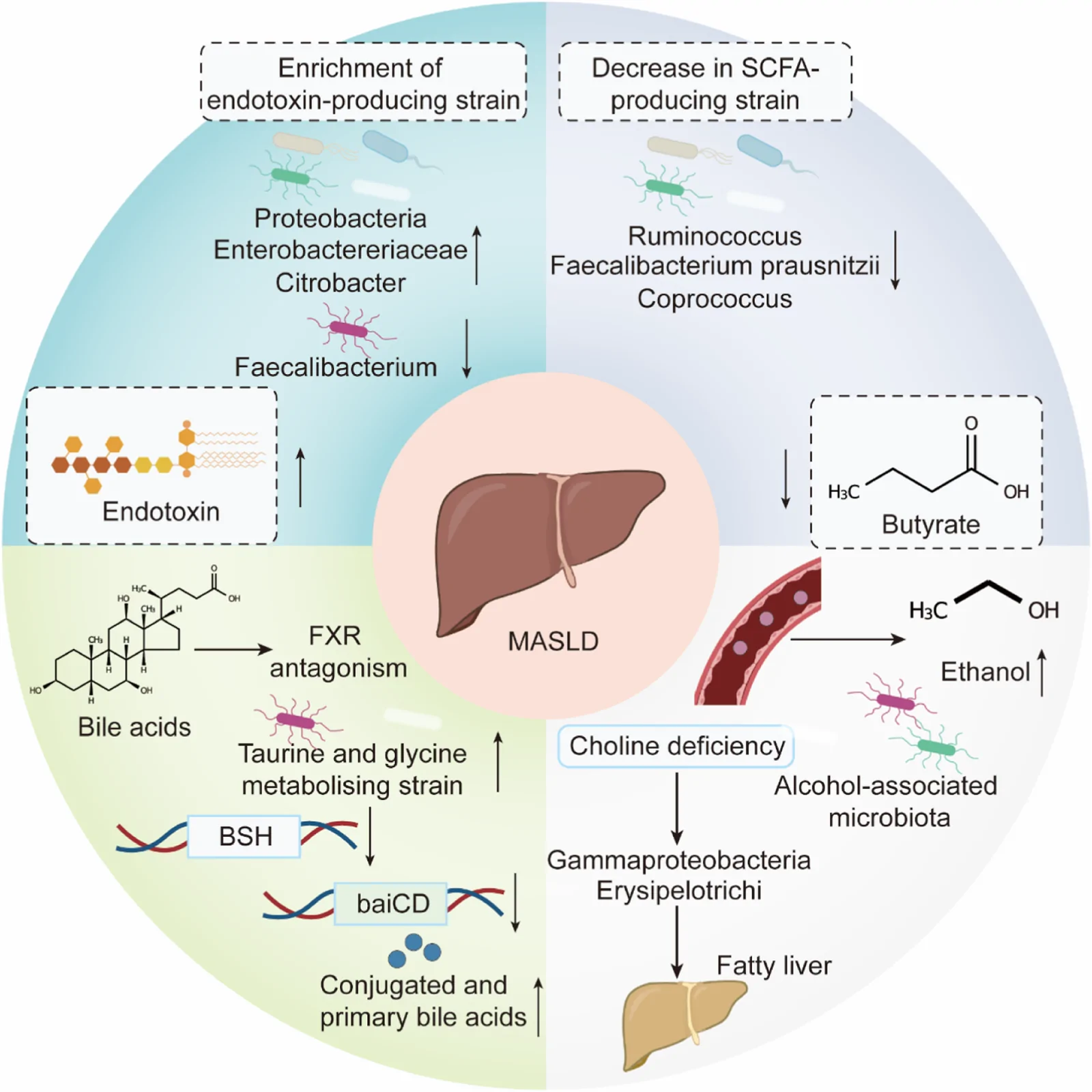
Gut microbial dysbiosis and associated metabolic alterations in MASLD. Gut dysbiosis in MASLD is initially characterized by a reduction in short-chain fatty acid-producing taxa. Levels of *Ruminococcus*, *Faecalibacterium prausnitzii*, and *Coprococcus* are decreased, leading to an overall reduction in the potential for short-chain fatty acid production. In addition, MASLD is commonly associated with an enrichment of potentially endotoxin-producing bacteria, as reflected by increased abundance of *Proteobacteria*, *Enterobacteriaceae*, and *Citrobacter*, which often occurs concurrently with a decline in *Faecalibacterium*. Beyond alterations related to short-chain fatty acids and endotoxin production, MASLD also involves abnormalities in bile acid-transforming microbial communities, characterized by increased levels of the FXR-antagonistic bile acid DCA and decreased levels of the FXR-agonistic bile acid CDCA, together with an increase in taurine- and glycine-metabolizing bacteria. Furthermore, MASLD is accompanied by downregulation of microbial genes involved in bile acid metabolism, including *bsh* and *baiCD*, as well as increased total conjugated bile acids and unconjugated primary bile acids. In patients with NASH, alcohol-associated gut microbes are also increased and are accompanied by elevated blood ethanol levels. With respect to choline metabolism, controlled-feeding human studies have shown that, under choline-deficient conditions, interindividual variation in *Gammaproteobacteria* and *Erysipelotrichi* is associated with the extent of hepatic fat accumulation, suggesting that the gut microbiota may influence susceptibility to fatty liver by altering choline availability.

From a functional perspective, MASLD-related dysbiosis often discussed in relation to SCFA-producing capacity, but this axis should be interpreted as compartment-specific rather than uniformly protective or uniformly depleted (Thing et al., 2024) (Figure 1). Human measurements have shown that total luminal SCFA concentrations decline from the caecum toward the descending colon, with carbohydrate fermentation occurring predominantly in the caecum and ascending colon (Cummings et al., 1987). Consistent with this regional gradient, SCFA production by caecal contents was reported to be up to eightfold higher than that by sigmoid or rectal contents, whereas products of protein fermentation progressively increased toward the distal colon, indicating that microbial metabolic output changes substantially along the colonic axis (Macfarlane et al., 1992). Regional SCFA concentrations should not, however, be equated directly with microbial production, because SCFAs are absorbed and metabolized by the colonic mucosa, such that the amount detected at a given site reflects the balance between local generation and host uptake (McNeil et al., 1978; Høverstad et al., 1982). This distinction is particularly important when comparing studies based on different sampling approaches. In a human crossover study, fibre supplementation enhanced distal colonic fermentation and increased plasma SCFA concentrations without producing a corresponding change in faecal SCFA content, demonstrating that stool measurements may fail to capture region-specific changes in fermentation (So et al., 2023). A subsequent isotope-tracer study similarly found that plasma, but not faecal, SCFA concentrations correlated with estimated intestinal SCFA production, further cautioning against interpreting faecal concentrations as a direct measure of microbial output (Kirschner et al., 2025). Thus, apparently discordant findings across MASLD studies may partly reflect differences in the intestinal segment and biological compartment sampled rather than genuine disagreement regarding microbial SCFA-producing capacity. A cross-sectional study of biopsy-confirmed adult NAFLD showed that Ruminococcus, *Faecalibacterium prausnitzii*, and Coprococcus were all reduced in both simple steatosis and NASH compared with healthy controls, and these differences were independent of body mass index (BMI) and insulin resistance, supporting the need to assess microbial changes beyond obesity alone (Da Silva et al., 2018). A Korean MAFLD cohort further demonstrated that the decline in Faecalibacterium was accompanied by an overall reduction in butyrate-producing bacteria, placing the emphasis on a broader reduction in butyrate-producing ecological functions rather than on a single depleted taxon (Oh et al., 2021). This finding is also consistent with a meta-analysis showing persistent reductions in Faecalibacterium, Ruminococcus, and Coprococcus (Cai et al., 2024).

Conversely, MASLD is also characterized by enrichment of potentially endotoxin-producing or proinflammatory taxa. Patients with MAFLD in the Korean cohort exhibited increased Proteobacteria, Enterobacteriaceae, and *Citrobacter*, occurring simultaneously with reduced Faecalibacterium, a pattern in which impaired protective anaerobic metabolic functions often coexist with expansion of opportunistic pathogens (Oh et al., 2021). A pediatric NASH study likewise found increases in Proteobacteria, Enterobacteriaceae, and *Escherichia*, and further showed that blood ethanol levels were higher in patients with NASH than in healthy controls and children with simple obesity, linking expansion of these taxa with both proinflammatory features and increased gut-derived metabolic toxins. This microbial pattern has been linked to increased endotoxin exposure (Zhu et al., 2013). For example, patients with NASH show both increased Enterobacteriaceae and elevated serum endotoxin levels, and Enterobacteriaceae abundance is even higher in those with more advanced fibrosis (Özkul et al., 2017). Beyond these metabolic and inflammatory consequences, the limited clinical literature has associated NAFLD with an increased risk of *Clostridioides difficile* infection and related intestinal complications (Kiseleva et al., 2023).

Beyond alterations in SCFAs and endotoxin-related functions, abnormalities in bile acid-transforming microbial communities have also been reported in MASLD. In addition, pediatric NASH studies have shown enrichment of alcohol-producing microbial taxa together with elevated blood ethanol levels, linking endogenous ethanol production with the pediatric NASH microbiome phenotype (Zhu et al., 2013). Microbial choline metabolism represents another recurrent functional alteration in MASLD-associated dysbiosis. In a controlled choline-depletion study, interindividual differences in Gammaproteobacteria and Erysipelotrichi were associated with the extent of hepatic fat accumulation, although this evidence is most directly relevant to diet-dependent susceptibility rather than to all spontaneous MASLD phenotypes (Spencer et al., 2011).

Taken together, MASLD-associated dysbiosis is characterized not only by altered microbial composition, but also by functional remodeling of microbial metabolism. Thus, microbial dysbiosis and intestinal barrier dysfunction should be understood as interconnected processes rather than separate events. The following section therefore examines how disruption of the intestinal mechanical, vascular, chemical, and immune barriers along the gut-liver axis amplifies microbial translocation and drives MASLD progression.

### 2.2 Intestinal barrier dysfunction along the gut–liver axis

Impairment of intestinal barrier function is a key event in the initiation and progression of MASLD (Gupta et al., 2022). The intestinal mechanical barrier is primarily composed of the intestinal mucosa, epithelial cells, and their junctional complexes, among which disruption of tight junction and adherens junction proteins constitutes a major basis for barrier instability (Gupta et al., 2022). Studies have shown that the expression of zonula occludens-1 (ZO-1) and occludin is reduced in intestinal epithelial cells from patients, and that this reduction is associated with elevated transaminase levels (Xin et al., 2014). In mice fed a high-fat diet, abnormalities in F11r, which encodes junctional adhesion molecule A (JAM-A), can further compromise epithelial integrity and are accompanied by increased colonic inflammatory proteins and the development of steatohepatitis (Rahm et al., 2016). As epithelial continuity is disrupted, intestinal permeability increases, allowing bacteria and their metabolites to cross the intestinal wall more readily and enter the circulation (Liu et al., 2023).

In addition to the epithelial barrier, impairment of the gut vascular barrier also promotes systemic translocation of bacteria and bacterial products (Spadoni et al., 2015). This change can occur as early as the initial stages of NASH and is associated with dysregulation of endothelial WNT/β-catenin signaling. With respect to the intestinal chemical barrier, bile acids and their enterohepatic circulation are also markedly altered in fatty liver disease (Mouries et al., 2019). Patients exhibit elevated serum bile acid levels that correlate with the degree of steatosis, whereas downregulation of FXR further weakens bile acid homeostasis and metabolic regulation, thereby aggravating gut-liver axis dysfunction (Bechmann et al., 2013; Jiao et al., 2018). Meanwhile, the intestinal immune barrier also contributes to disease progression, as reflected by reduced regulatory T cells (Tregs) and increased T helper 1 (Th1) cells and CD8^+^ T cells in the intestinal lamina propria, indicating impaired local immune tolerance (Gupta et al., 2022). In addition, mast cell-derived tryptase, chymase, histamine, and various cytokines can degrade ZO-1, downregulate JAM-A, and increase epithelial permeability (Wilcz-Villega et al., 2013; Scudamore et al., 1998). When portal hypertension is present, it may further amplify barrier disruption by aggravating intestinal mucosal edema, widening intercellular spaces, and increasing microvascular permeability, accompanied by elevated LBP and IL-6, suggesting propagation of gut-derived inflammatory signals to the liver (Reiberger et al., 2013; Sorribas et al., 2019).

On the basis of sequential injury across multiple barrier layers, gut dysbiosis further amplifies intestinal barrier damage and hepatic inflammation (Sorribas et al., 2019). Taken together, impairment of the intestinal mechanical, vascular, chemical, and immune barriers in MASLD is not independent of microbial dysbiosis; rather, these processes mutually reinforce one another and collectively drive the transmission of gut-derived inflammation and metabolic disturbance to the liver (Gupta et al., 2022). Existing studies also suggest that probiotics, prebiotics, and FMT may improve intestinal permeability or certain metabolic parameters to some extent (Gupta et al., 2022; Kobyliak et al., 2018).

## 3 Functional mechanisms linking gut dysbiosis to MASLD progression

Gut dysbiosis influences MASLD progression first through alterations in the microbial metabolite profile, including short-chain fatty acids, choline-related TMA/TMAO, endogenous ethanol, LPS, and bile acids. Through the gut-liver axis, these signals can interfere with hepatocellular lipid export, insulin sensitivity, bile acid receptor signaling, and inflammatory responses, although the direction and magnitude of their effects often depend on sample type, disease stage, and experimental model (Thing et al., 2024; Romano et al., 2015; Tan et al., 2019; Gan et al., 2023; Soppert et al., 2023). Beyond direct metabolic effects, the gut microbiota and its products can also amplify intrahepatic inflammation by disrupting the intestinal barrier, increasing antigen exposure, and activating immune cells. For example, studies in mouse models of NASH have shown that microbiota-related signals enhance B-cell antigen-presenting capacity and proinflammatory function, thereby further driving T-cell inflammation and fibrotic responses (Barrow et al., 2021; Kotsiliti et al., 2023). At a deeper regulatory level, microbial signals may also participate in host epigenetic reprogramming. An allogeneic FMT study suggested that microbiota remodeling can be accompanied by changes in hepatic DNA methylation in patients with NAFLD, whereas experiments in high-fat, high-cholesterol diet-induced MASLD mice and hepatocytes showed that Phocaeicola vulgatus-derived 3-HPAA attenuates lipid deposition by reducing H3K27 acetylation and SQLE transcription (Stols-Gonçalves et al., 2023; Jin et al., 2024). Accordingly, this section discusses the functional mechanisms linking gut dysbiosis to MASLD from three interconnected perspectives: microbial metabolites, immune dysregulation, and epigenetic regulation.

### 3.1 Microbiota-derived metabolic pathways in MASLD

#### 3.1.1 Compartment-specific SCFA and BCFA dysregulation in MASLD

**FIGURE 2:**
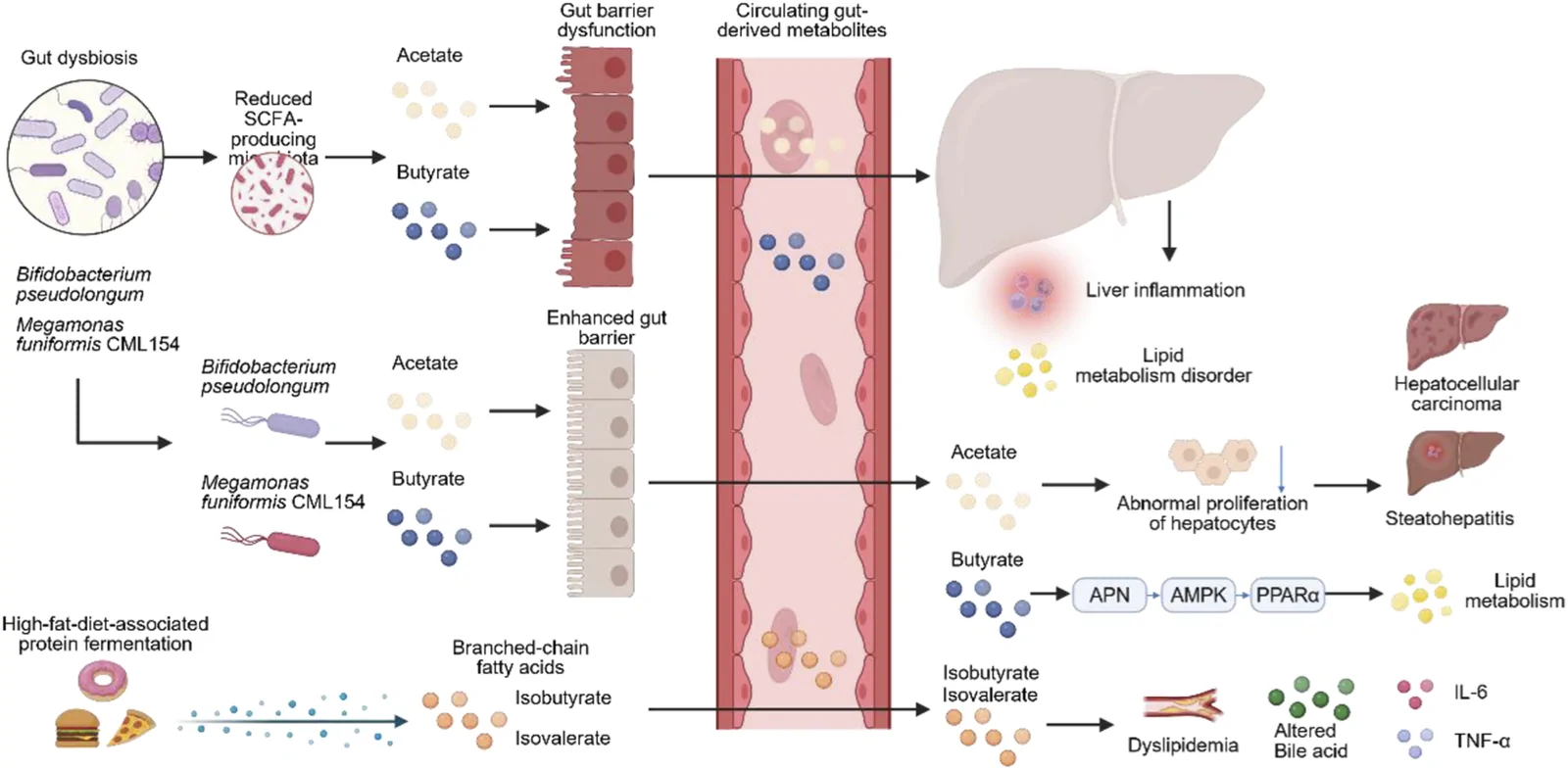
Short-chain fatty acid dysregulation in MASLD and MASH-related liver injury. A reduction in short-chain fatty acid-producing bacteria decreases acetate and butyrate levels, compromising intestinal barrier stability and aggravating hepatic inflammation and lipid metabolic dysfunction. Acetate derived from *Bifidobacterium pseudolongum* enhances intestinal barrier function and suppresses aberrant hepatocyte proliferation, thereby attenuating steatohepatitis and MASH-associated hepatocellular carcinoma development. *Megamonas funiformis* CML154 increases intestinal propionate levels and improves lipid metabolism through the APN-AMPK-PPARα signaling pathway. In contrast, enhanced high-fat diet-associated protein fermentation is accompanied by increased isobutyrate and isovalerate levels, which are associated with dyslipidemia, altered bile acid profiles, and increased hepatic IL-6 and TNF-α expression.

SCFAs are important metabolites generated through the gut microbial fermentation of indigestible proteins and dietary fibers, and they play key roles in regulating immune responses and maintaining intestinal barrier integrity. In patients with MASLD, the abundance of several SCFA-producing bacterial taxa is reduced. Compared with healthy controls (n = 50), patients with MASLD (n = 100) generally show lower plasma acetate levels, whereas other SCFAs, including propionate, are increased and associated with liver fibrosis (Figure 2) (Thing et al., 2024). Among these metabolites, propionate appears to be particularly associated with greater fibrosis severity and may also contribute to insulin resistance in humans (Thing et al., 2024; Tirosh et al., 2019). However, findings from fecal samples do not fully recapitulate those derived from plasma analyses. A 2025 cross-sectional study including 33 patients with NAFLD used GC-MS to quantify fecal acetate, propionate, and butyrate, and reported an acetate:propionate:butyrate ratio of 59:24:17. In that study, absolute fecal butyrate levels showed a moderate inverse correlation with the controlled attenuation parameter value (r = −0.522, p = 0.002), whereas fecal SCFA levels were not associated with transient elastography measurements (Astari et al., 2025). In addition, propionate-related findings are not uniform across animal studies. In an HFD-induced MAFLD study using avian and mouse models, inulin-enriched Megamonas was negatively associated with hepatic steatosis-related parameters. Oral administration of the isolated strain M. funiformis CML154 to HFD-fed animals ameliorated MAFLD, altered hepatic gene-expression profiles, and increased intestinal propionate levels. The authors attributed its anti-MAFLD effects to propionate-mediated activation of the APN-AMPK-PPARα signaling pathway (Yang et al., 2023). Conversely, acetate produced by Bifidobacterium pseudolongum can protect against diet-induced steatohepatitis and MASH-associated hepatocellular carcinoma by enhancing intestinal barrier stability and directly suppressing hepatocyte proliferation (Song et al., 2023).

Beyond SCFAs, protein fermentation can also generate other metabolites, including branched-chain fatty acids (BCFAs), mainly isobutyrate and isovalerate. BCFA production is influenced by dietary quality, and fecal BCFA levels are associated with lower intake of dietary insoluble fiber (Rios-Covian et al., 2020). Compared with control individuals, patients with MASLD and MASH have higher total hepatic BCFA levels. In patients with MASLD, total hepatic BCFA levels correlate with disease severity (Martínez-Montoro et al., 2023). A recent multi-omics study in mice also examined intestinal SCFAs and BCFAs in a MASLD model. In that study, HFD-induced MASLD mice showed reduced total fecal SCFAs, with acetate, propionate, and butyrate all lower than those in normal diet-fed controls, whereas isobutyrate and isovalerate were increased. Mediterranean diet intervention significantly increased propionate levels and reduced isobutyrate and isovalerate levels (Wan et al., 2025). In the correlation analysis, isobutyrate and isovalerate were positively correlated with body weight, epididymal white adipose tissue weight, total bile acids, serum total cholesterol, triglycerides, AST, and hepatic IL-6 and TNF-α. In contrast, acetate, propionate, and butyrate were negatively correlated with fasting blood glucose, serum total cholesterol, ALT, and hepatic TNF-α (Wan et al., 2025). In a biopsy-defined case-control study, hepatic BCFAs were elevated and correlated with histopathological and biochemical features of NAFLD, whereas serum BCFAs showed neither group differences nor disease-related correlations (Martínez-Montoro et al., 2023). Lower circulating isobutyrate and methylbutyrate were associated with greater ultrasound-defined NAFLD severity in patients with type 2 diabetes (Tsai et al., 2023), whereas another biopsy-characterized MASLD cohort found no overall association of plasma isobutyrate or isovalerate with MASLD; isobutyrate was inversely associated with severe steatosis but was higher in F4 than in F0 fibrosis (Thing et al., 2024). These discordant cross-sectional findings indicate that BCFAs remain exploratory biomarkers and require standardized longitudinal validation before their diagnostic or prognostic value can be established.

#### 3.1.2 Gut-derived ethanol and microbial drivers of steatohepatitis

Ethanol is a by-product of bacterial and fungal fermentation and can also be produced endogenously in humans. Under physiological conditions, ethanol may be metabolized by enzymes in intestinal epithelial cells or, after entering the circulation, transported through the portal vein to the liver for further metabolism (Meijnikm et al., 2024). Disruption of intestinal homeostasis may increase microbial ethanol production. Previous studies have shown that patients with MASH have elevated peripheral blood alcohol levels, with even higher ethanol concentrations in the portal circulation, and that this increase becomes more pronounced with increasing liver disease severity (Zhu et al., 2013; Meijnikman et al., 2022; Engstler et al., 2016). A study further showed that, among 146 participants, median portal venous ethanol concentrations were 187-fold higher than fasting peripheral venous levels and increased with disease progression, from 2.1 mM in individuals without steatosis to 8.0 mM in patients with NAFL and 21.0 mM in patients with NASH. The portal ethanol concentration of 21.0 mM corresponds to a substantial hepatic ethanol exposure, but the marked portal-to-peripheral gradient suggests extensive first-pass hepatic clearance (Meijnikman et al., 2022). Whether this exposure alone is sufficient to promote liver injury remains uncertain, as its duration and direct contribution to disease progression were not assessed (Meijnikman et al., 2022). In an intervention study, selective inhibition of alcohol dehydrogenase (ADH) increased peripheral blood ethanol concentrations by 15-fold in patients with NAFLD, whereas this effect disappeared after antibiotic treatment (Meijnikman et al., 2022). In rare extreme cases, excessive microbially derived ethanol production may exceed hepatic metabolic capacity, leading to abnormally elevated blood alcohol levels and the development of auto-brewery syndrome (Yuan et al., 2019). A recent case-control study of auto-brewery syndrome further showed that the abundance of *Klebsiella* in the gut was closely associated with fluctuations in blood alcohol concentrations. The investigators isolated three high-alcohol-producing *Klebsiella* species and confirmed that these strains could induce auto-brewery syndrome in mice (Xue et al., 2023).

**FIGURE 3:**
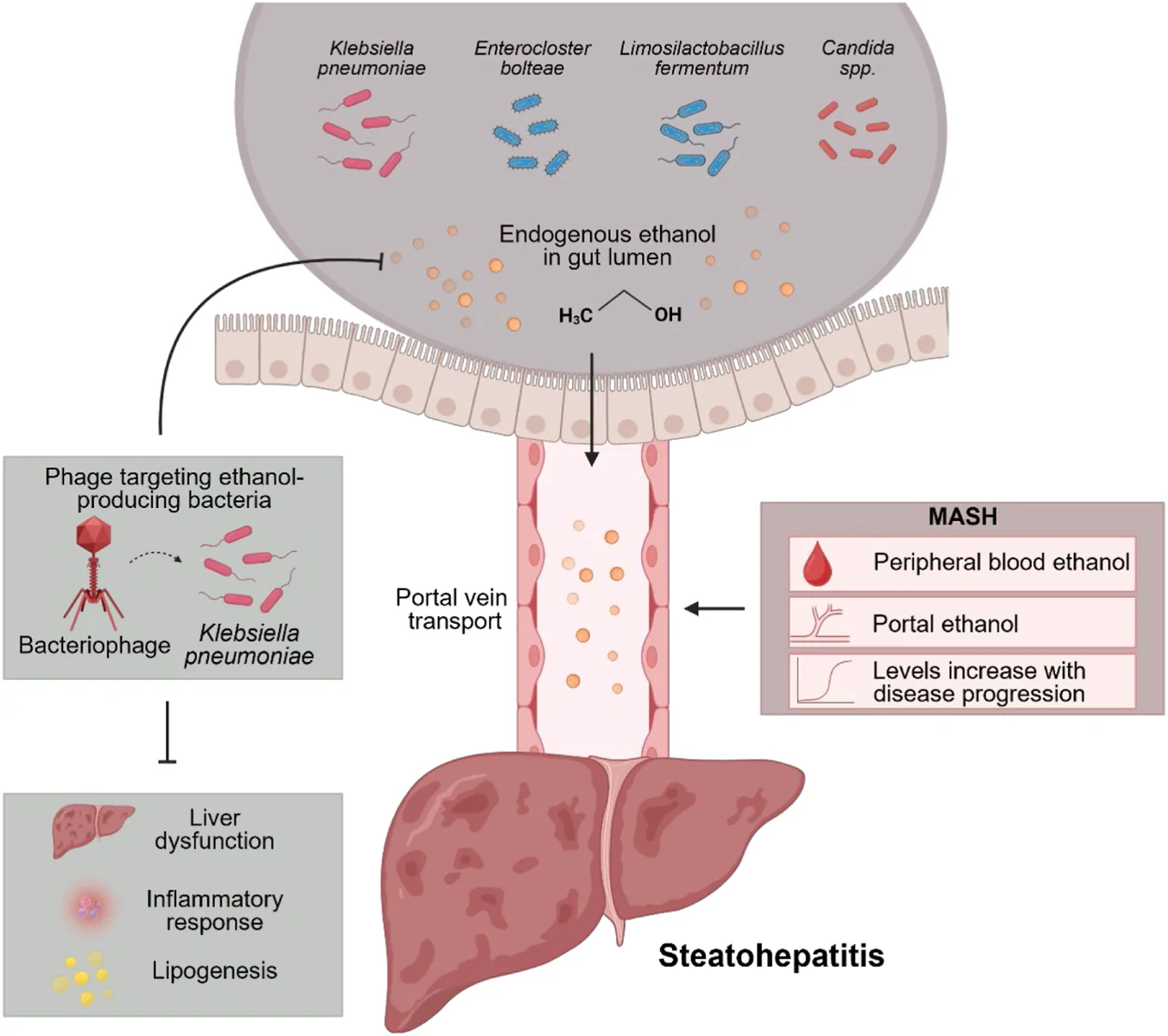
Microbial ethanol production drives gut-liver axis injury in MASH. After entering the liver through the portal vein, ethanol exacerbates hepatocellular lipid accumulation, inflammatory cytokine expression, and steatohepatitis progression. Patients with MASH show elevated ethanol levels in both peripheral blood and portal circulation, and these levels increase with disease progression. High-alcohol-producing *Klebsiella pneumoniae* represents a well-defined functional strain that can induce steatohepatitis, whereas bacteriophages targeting ethanol-producing bacteria can attenuate liver injury, inflammatory responses, and lipogenesis. *Enterocloster bolteae*, *Limosilactobacillus fermentum*, and certain *Candida* species may also serve as ethanol sources and contribute to NASH-associated gut-liver axis injury.

A study in which fecal microbiota from MASH patients carrying ethanol-producing *Klebsiella pneumoniae* were transplanted into mice demonstrated that this microbial community could induce steatohepatitis, whereas intervention with bacteriophages targeting ethanol-producing bacteria attenuated this pathological process (Gan et al., 2023). In a male mouse model of steatohepatitis induced by high-alcohol-producing *Klebsiella pneumoniae* (HiAlc Kpn), HiAlc Kpn-specific bacteriophages alleviated liver function abnormalities, inflammatory cytokine expression, and lipogenic gene expression, without causing evident pathological alterations in liver or kidney function or in gut microbiota composition (Figure 3) (Gan et al., 2023). Other gut bacteria also have ethanol-producing capacity and may contribute to hepatic inflammatory responses (Purohit et al., 2024). A 2023 case-control study including 41 patients with NASH and 24 healthy controls found that fecal ethanol and glucose levels were elevated in patients with NASH. Culturomics analysis showed enrichment of the ethanol-producing bacteria *Enterocloster bolteae* and *Limosilactobacillus fermentum* in NASH, while V3-V4 16S rRNA amplicon sequencing further identified enrichment of ethanol-producing taxa, including *L. fermentum*, *Mediterraneibacter gnavus*, and *Streptococcus mutans* (Mbaye et al., 2023). In addition, gut fungi may also contribute to endogenous ethanol production; one NASH study reported that intestinal yeasts such as *Pichia kudriavzevii*, *Candida albicans*, and *Candida glabrata* were capable of producing ethanol and triglycerides (Mbaye et al., 2022). Collectively, these findings support a link between ethanol-producing microorganisms and steatohepatitis, although this mechanism is unlikely to apply uniformly across all patients with MASLD. High-alcohol-producing *Klebsiella pneumoniae* was associated with up to 60% of individuals with NAFLD in a Chinese cohort, supporting its relevance to a substantial but incomplete patient subgroup (Yuan et al., 2019). Moreover, different ethanol-producing taxa were enriched in another NASH cohort, suggesting that this phenotype is not attributable to a single conserved microorganism (Mbaye et al., 2023). Microbiota-derived ethanol may therefore be particularly relevant in patients harboring ethanol-producing microbial communities, although the prevalence and contribution of this phenotype across broader MASLD populations remain to be established.

#### 3.1.3 Choline metabolism

In the liver, choline is converted into phosphatidylcholine, an essential structural component required for very low-density lipoprotein (VLDL) assembly and secretion. In primary hepatocytes isolated from choline-deficient rats, supplementation with either choline or methionine restored the secretion of VLDL-associated triglycerides and phosphatidylcholine, consistent with the requirement for active phosphatidylcholine synthesis in hepatic VLDL secretion and triglyceride export (Yao and Vance, 1988). In a controlled feeding study in humans, dietary choline deprivation induced fatty liver or muscle injury in 77% of men and 80% of postmenopausal women. Consistent with this, phosphatidylethanolamine N-methyltransferase-deficient (Pemt^−/−^) mice exhibited reduced hepatic phosphatidylcholine levels despite receiving the recommended amount of choline, accompanied by steatosis and an increase in terminal deoxynucleotidyl transferase dUTP nick end labeling (TUNEL)-positive cells. Together, these data place host phosphatidylcholine synthesis capacity upstream of microbial choline competition: reduced dietary choline intake, menopausal status, and impaired PEMT-dependent phosphatidylcholine synthesis can each increase vulnerability to hepatic lipid accumulation (Fischer et al., 2007; Zhu et al., 2003). Furthermore, in human populations, the loss-of-function PEMT V175M variant has been associated with susceptibility to NAFLD, providing genetic support for the concept that reduced endogenous phosphatidylcholine compensatory capacity increases host dependence on exogenous choline supply (Fischer et al., 2007; Zhu et al., 2003; Song et al., 2005). Evidence linking PEMT to NAFLD remains mixed, as subsequent genetic studies and meta-analyses reported inconsistent associations between PEMT rs7946/V175M and NAFLD risk in different populations (Song et al., 2005; Dong et al., 2007; Tan et al., 2016).

Against this background of increased reliance on exogenous choline, microbial choline metabolism is more likely to influence hepatic lipid homeostasis. When 129S6 mice developed fatty liver under a high-fat diet, they exhibited reduced plasma phosphatidylcholine together with increased urinary dimethylamine, trimethylamine (TMA), and trimethylamine N-oxide (TMAO), linking high-fat diet-induced fatty liver in this strain to microbial methylamine production and reduced host-accessible choline (Dumas et al., 2006). In a 2-month inpatient controlled-feeding study, fecal microbial composition in 15 women changed in response to dietary choline levels during choline depletion, and interindividual differences in *Gammaproteobacteria* and *Erysipelotrichi* were directly associated with changes in liver fat, indicating that under the same choline-restricted condition, microbial composition can modulate host susceptibility to hepatic lipid accumulation (Spencer et al., 2011).

At the molecular level, Craciun et al. identified the choline utilization gene cluster in the sulfate-reducing bacterium *Desulfovibrio desulfuricans* and demonstrated that CutC functions as a choline TMA-lyase, whereas CutD serves as its activating protein. This system cleaves choline into TMA and acetaldehyde (Craciun and Balskus, 2012). Subsequently, Romano et al. further showed in colonized mice carrying human gut isolates that different microbial community structures vary in their capacity to generate TMA and TMAO and to affect host choline bioavailability. Even low-level colonization by TMA-producing bacteria was sufficient to reduce host-accessible choline (Romano et al., 2015). Compared with genus-level dysbiosis, these studies provide a more mechanistic layer of evidence because they identify the enzyme system and colonization-dependent functional capacity required for TMA formation (Romano et al., 2015; Craciun et al., 2014). In a further study of human fecal samples, Rath et al. detected cutC in all 50 samples examined, but its abundance was below 1% of the total microbial community in most individuals. They also showed that 16S ribosomal RNA (rRNA) sequencing alone is insufficient to infer the TMA-producing potential of a microbial community, suggesting that the risk of choline depletion should be evaluated at the level of functional genes rather than inferred directly from genus-level compositional changes (Rath et al., 2017). Because cutC was widely detectable but usually low in relative abundance, taxonomic shifts alone cannot be equated with increased microbial choline consumption unless functional-gene abundance or direct TMA/TMAO production is measured (Rath et al., 2017).

After microbial conversion of choline into TMA, TMA can be further oxidized in the liver by flavin-containing monooxygenase 3 (FMO3) into TMAO (Bennett et al., 2013). For this reason, circulating TMAO should be interpreted as a composite readout of dietary substrate supply, microbial TMA-producing capacity, and hepatic FMO activity rather than as a microbiota-only marker (Romano et al., 2015; Zhu et al., 2018). Clinical studies have shown that increased serum TMAO is associated with both the presence and severity of NAFLD (Chen et al., 2016). In patients with biopsy-confirmed NAFLD, elevated TMAO was associated with increased total bile acid levels, a higher proportion of farnesoid X receptor (FXR)-antagonistic bile acids, and increased hepatic CYP7A1 messenger RNA (mRNA). In parallel, in high-fat diet-fed mice and palmitate-treated HepG2 cells, TMAO promoted hepatic lipid accumulation by enhancing CYP7A1-related bile acid synthesis and suppressing FXR signaling (Tan et al., 2019). Together, these data connect microbiota-mediated choline metabolism not only with reduced host choline availability but also with TMAO-related bile acid remodeling and FXR suppression in experimental NAFLD models (Tan et al., 2019; Bennett et al., 2013). Because the cited human studies were case-control or cross-sectional, the human evidence is best framed as an association between choline-related metabolites and NAFLD features, whereas the strongest causal evidence still comes from controlled dietary, gnotobiotic, and cell or mouse experiments (Spencer et al., 2011; Romano et al., 2015; Tan et al., 2019; Chen et al., 2016).

This association in human populations has been further supported by animal studies. In a cross-sectional analysis of 664 subjects with biopsy-confirmed NAFLD, inadequate choline intake in postmenopausal women was associated with more severe fibrosis (Guerrerio et al., 2012). A high-fat diet-induced MASLD mouse study further showed that intestinal IL-33 promotes disease progression by increasing TMAO-producing bacteria and intestinal permeability, whereas intestinal IL-33 deficiency reduced both, and inhibition of TMAO synthesis with 3,3-dimethyl-1-butanol attenuated hepatic oxidative stress (Hai et al., 2025). The IL-33 study adds an inflammatory and barrier-related layer to this pathway, but it tests a defined high-fat diet mouse model and does not establish TMAO as the dominant mediator across all human MASLD phenotypes (Hai et al., 2025). Overall, choline metabolism is one of the more mechanistically traceable microbiota-related pathways in MASLD: host choline demand depends on dietary supply and PEMT-dependent phosphatidylcholine synthesis, microbial cutC-dependent metabolism can divert choline toward TMA, hepatic FMO3 converts TMA into TMAO, and TMAO can interact with bile acid-FXR signaling in experimental systems; the main limitation is that human evidence still relies largely on association, small controlled-feeding cohorts, and population-specific genetic data.

#### 3.1.4 Insulin resistance and metabolic endotoxaemia

In high-fat diet-fed mice, circulating lipopolysaccharide (LPS) increased approximately two- to threefold within 4 weeks, suggesting that metabolic endotoxemia is an early event in diet-induced metabolic dysfunction. Accordingly, continuous low-dose LPS infusion for 4 weeks was sufficient to induce fasting hyperglycemia, hyperinsulinemia, hepatic triglyceride accumulation, and hepatic insulin resistance, indicating that microbiota-derived LPS can directly contribute to the development of MASLD-related metabolic phenotypes (Cani et al., 2007). However, subsequent study showed that 8 weeks of high-fat diet feeding induced obesity and increased plasma LPS-binding protein (LBP) levels in mice, whereas body weight gain and fat accumulation were not attenuated in *Tlr4* ^−/−^ or *Cd14* ^−/−^ mice. These findings suggest that high-fat diet-induced obesity and adiposity are not necessarily dependent on TLR4 or CD14 signaling (Dalby et al., 2018).

In high-fat diet-fed and ob/ob mice, antibiotic treatment reduced cecal LPS content and circulating endotoxin levels, accompanied by improved glucose tolerance and attenuation of adipose tissue inflammation and oxidative stress. Further studies showed that a high-fat diet itself increased intestinal permeability and downregulated tight junction-related genes, whereas CD14 deficiency partially reproduced the metabolic protective effects of antibiotics, suggesting that microbiota-derived LPS contributes to insulin resistance through CD14-mediated recognition (Cani et al., 2008). However, in human studies, TLR4 blockade has not consistently reproduced this metabolic protection. Short-term administration of eritoran, a competitive TLR4 inhibitor, failed to prevent lipid infusion-induced insulin resistance and did not improve basal insulin resistance in individuals with obesity (Liang et al., 2022). Oligofructose-driven remodeling of the gut microbiota, marked by increased *Bifidobacterium* spp., *Lactobacillus* spp., and the *Clostridium coccoides*/*Eubacterium rectale* cluster, enhanced endogenous GLP-2 production, strengthened intestinal tight junctions, reduced endotoxaemia, and consequently alleviated hepatic inflammation and oxidative stress in ob/ob mice (Cani et al., 2009). At the human level, a study of 155 patients with NAFLD showed that serum endotoxin levels were significantly higher than those in healthy controls and were positively correlated with insulin resistance (Harte et al., 2010). A systematic review and meta-analysis including 34 observational studies further showed that blood endotoxin levels were overall higher in patients with NAFLD than in liver-healthy controls, with elevations observed in both simple steatosis and NASH. However, this study also noted substantial heterogeneity in findings based on LBP as an indirect endotoxin marker, and EndoCab IgG showed no association with histological signs of liver injury (Soppert et al., 2023). In addition, among adults with obesity stratified by liver histology, endotoxin-related markers, including LPS, LBP, and sCD14, did not distinguish individuals with normal liver histology from those with NAFL or NASH. In that study, disease severity in NAFLD was associated with pro-inflammatory mediators such as IL-8, TNF-α, and CCL3, rather than endotoxin-related parameters (du Plessis et al., 2016). Notably, in early pediatric NAFLD, elevations in endotoxin and LPS-binding protein (LBP) were already detectable and were mainly associated with inflammatory indices, whereas no association with insulin resistance parameters was observed, suggesting that endotoxin-related inflammatory changes may emerge at an early stage of disease (Nier et al., 2017). Similarly, a study including adolescents with obesity, with or without biopsy-confirmed MASLD, showed that EndoCab IgG, LBP, and CRP were elevated in both the MASLD and simple obesity groups compared with the normal-weight group. However, these markers were not further increased in the MASLD group compared with the simple obesity group, and endotoxin markers did not vary according to sex, steatosis grade, or fibrosis stage (Boone et al., 2026). Overall, current evidence supports the involvement of LPS-related metabolic endotoxemia in inflammation and metabolic dysregulation in MASLD, but its causal role in humans and its relationship with insulin resistance and disease progression remain uncertain. Future studies incorporating histological stratification and longitudinal interventions are needed to clarify the causal contribution and clinical targetability of endotoxin exposure in MASLD.

#### 3.1.5 Bile acid signaling

After being synthesized in the liver and secreted into the intestinal lumen, bile acids undergo further transformation in the gut, a process that is highly dependent on microbial enzymatic systems. BSH-mediated deconjugation serves as the entry step for subsequent microbial conversion, whereas 7α-dehydroxylating bacteria such as *Clostridium scindens* further convert primary bile acids into secondary bile acids such as deoxycholic acid (DCA) and lithocholic acid (LCA) through the bai operon (Jones et al., 2008; Ridlon and Hylemon, 2012). Mouzaki et al. compared healthy controls with patients with nonalcoholic fatty liver (NAFL) and NASH and found that patients with NASH exhibited reduced Bacteroidetes and *Clostridium leptum*. Moreover, *C. leptum* was positively correlated with fecal free LCA and negatively correlated with free cholic acid and chenodeoxycholic acid (Mouzaki et al., 2016).

In a human study stratified by liver biopsy findings in 122 individuals, both serum and fecal bile acids progressively increased as NAFLD advanced from no or mild fibrosis to stage F3/F4 fibrosis. The increase in serum bile acids was mainly driven by conjugated primary bile acids, whereas the fecal increase was dominated by free secondary bile acids represented by DCA. In addition, serum glycocholic acid and fecal DCA were associated with the abundance of Bacteroidaceae and Lachnospiraceae, respectively (Adams et al., 2020). Further fecal multi-omics analyses showed that with increasing NAFLD activity and fibrosis severity, DCA-related metabolites, microbial baiCD and hdhA, and BSH expression were synchronously increased, whereas Ruminococcaceae, which are more sensitive to the antimicrobial effects of DCA, were decreased (Smirnova et al., 2022).

In high-fat diet-fed mice, tempol preferentially reduced *Lactobacillus* and its BSH activity, causing intestinal accumulation of tauro-β-muricholic acid and suppression of ileal FXR. In another study, either the intestine-restricted FXR antagonist Gly-MCA or intestinal epithelial-specific FXR deletion reduced serum ceramide and alleviated high-fat diet-induced metabolic dysfunction and fatty liver (Li et al., 2013; Gonzalez et al., 2016). In C57BL/6 mice fed a high-fat diet, CAPE alleviated hepatic steatosis by inhibiting gut microbial BSH activity, thereby increasing ileal tauro-β-muricholic acid, suppressing intestinal FXR signaling, and reducing ceramide production (Zhong et al., 2023). In another high-fat diet-fed mouse study, Astragaloside IV attenuated hepatic lipogenesis by remodeling gut microbiota-associated bile acid metabolism, suppressing intestinal FXR signaling, and lowering ceramide levels. FMT and intestinal epithelial-specific Fxr knockout experiments further supported the dependence of this effect on the gut microbiota-bile acid-FXR pathway (Zhai et al., 2022).

In addition to FXR, bile acids can also influence glucose metabolism through the Takeda G protein-coupled receptor 5 (TGR5)-mediated enteroendocrine pathway. In STC-1 cells, obese mice, and obese patients with diabetes, bile acids or TGR5 activation enhanced GLP-1 secretion and improved glucose homeostasis (Katsuma et al., 2005; Thomas et al., 2009; Calderon et al., 2020). In human and murine NASH liver tissue, TGR5 expression was reduced, whereas TGR5 deficiency enhanced NLR family pyrin domain containing 3 (NLRP3) inflammasome activation, caspase-1 cleavage, and M1 macrophage polarization, thereby aggravating hepatic steatosis and inflammation. These findings suggest that when microbial dysbiosis drives bile acid signaling away from homeostatic regulation toward imbalance, the consequences extend beyond lipid metabolic disturbance to further amplification of intrahepatic innate immune inflammation (Shi et al., 2020).

### 3.2 Immune dysregulation driven by the gut microbiota

**FIGURE 4:**
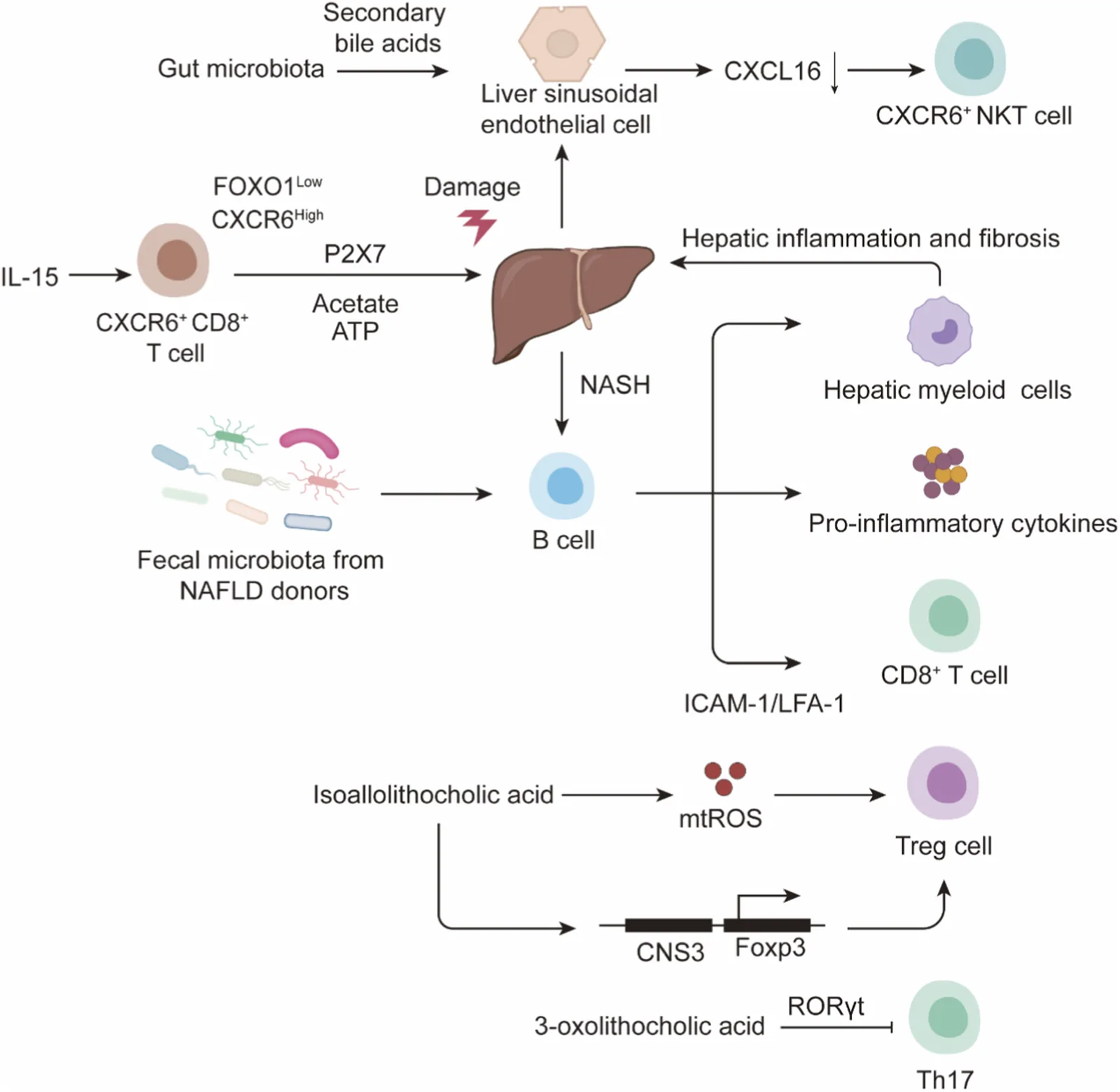
Regulation of B Cell, T Cell, and Innate-Like T Cell Responses by Gut Microbiota and Their Metabolites. Gut microbiota and their bile acid metabolites contribute to NASH-associated intrahepatic inflammatory injury by regulating B cell, T cell, and innate-like T cell responses. In NASH, hepatic B cells accumulate and exhibit enhanced pro-inflammatory cytokine secretion and antigen-presenting capacity; similarly, transplantation of fecal microbiota from human NAFLD donors into recipient mice promotes the accumulation and activation of hepatic B cells. B cells can also promote CD8^+^ T cell activation in the lamina propria through an ICAM-1/LFA-1-dependent mechanism and activate hepatic myeloid cells through the IgA–FcR axis, thereby aggravating intrahepatic inflammation and fibrosis. Meanwhile, microbiota-derived bile acid metabolites can directly regulate T cell differentiation. Among them, 3-oxolithocholic acid directly binds RORγt and suppresses Th17 differentiation, whereas isoallolithocholic acid promotes Treg differentiation by enhancing mitochondrial ROS in a Foxp3 CNS3-dependent manner. In addition, gut microbiota-mediated bile acid transformation regulates the expression of CXCL16 in liver sinusoidal endothelial cells, thereby influencing the accumulation of hepatic CXCR6^+^ NKT cells and their IFN-γ response. In CXCR6^+^CD8^+^ T cells, IL-15 first induces a FOXO1^low^, CXCR6^high^ state, after which acetate and extracellular ATP further trigger MHC-I-independent hepatocyte killing through P2X7.

In human NAFLD, the prevalence of small intestinal bacterial overgrowth is increased, intestinal permeability is elevated, and serum endotoxin or anti-endotoxin immunoglobulin G (IgG) levels are increased, with some of these indices showing a positive relationship with NASH activity and inflammation (Harte et al., 2010; Verdam et al., 2011). In histologically stratified human cohorts, hepatic transcriptional signatures in NASH are enriched for genes related to inflammatory responses, antigen presentation, and cytotoxic lymphocytes, accompanied by alterations in peripheral conventional dendritic cell type 1 (cDC1), cDC2, and CD8^+^ T-cell lineages. Single-cell transcriptomic analysis of 10 biopsy-confirmed NASH samples further revealed cell type-specific inflammatory and fibrotic programs within the liver (Haas et al., 2019; Fred et al., 2022). Further functional studies suggest that B cells may represent an important link through which the gut microbiota affects intrahepatic adaptive immunity. In a mouse NASH model induced by a high-fat, high-cholesterol diet, hepatic B cells accumulated and displayed enhanced proinflammatory cytokine secretion and antigen-presenting capacity (Figure 4). Either global B-cell deficiency or B cell-specific Myd88 deficiency alleviated hepatic T-cell inflammation and fibrosis, whereas transplantation of fecal microbiota from human NAFLD donors into recipient mice promoted NASH progression and increased hepatic B-cell accumulation and activation (Barrow et al., 2021). In cholate-deficient high-fat diet-induced mouse NASH and in human NASH samples, activated intestinal B cells expanded markedly, and depletion of either systemic or gastrointestinal B cells prevented or reversed NASH and liver fibrosis (Kotsiliti et al., 2023). Mechanistically, these B cells promoted CD8^+^ T-cell activation in the lamina propria through an intercellular adhesion molecule 1-lymphocyte function-associated antigen 1 (ICAM-1/LFA-1)-dependent manner and activated hepatic myeloid cells through the immunoglobulin A-Fc receptor (IgA-FcR) axis, thereby cooperatively aggravating intrahepatic inflammation and fibrosis (Kotsiliti et al., 2023). Despite these mechanistic findings, direct human evidence linking the gut microbiota to B-cell activation in MASLD remains limited. The microbiota-dependent pathway has been demonstrated primarily in mouse models, including transplantation of microbiota from patients with NAFLD into recipient mice (Barrow et al., 2021). Although activated intestinal B cells and IgA–FcR-related fibrotic responses were also observed in human NASH samples, intestinal B-cell activation in this study occurred independently of the gut microbiota, and a causal microbiota–B-cell relationship was not established in patients (Kotsiliti et al., 2023). Longitudinal and interventional human studies are therefore required to determine the contribution of microbiota-driven B-cell responses to MASLD progression.

In mice, acetate, propionate, and butyrate expand the colonic regulatory T-cell (Treg) pool (Smith et al., 2013; Furusawa et al., 2013). However, SCFAs do not necessarily exert exclusively immunosuppressive effects. In murine naïve CD4^+^ T cells, their effects depend on the specific polarization context; under both T helper 1 (Th1)- and T helper 17 (Th17)-inducing conditions, SCFAs can promote the corresponding effector differentiation while simultaneously enhancing interleukin-10 (IL-10) production (Park et al., 2015). In addition, bacterial secondary bile acid metabolites now have direct molecular evidence for regulating T-cell differentiation. In murine T-cell systems, 3-oxolithocholic acid can directly bind retinoic acid receptor-related orphan receptor gamma t (RORγt) and inhibit Th17 differentiation, whereas isoallolithocholic acid promotes Treg differentiation by enhancing mitochondrial reactive oxygen species (ROS) and depending on the conserved non-coding sequence 3 (CNS3) region of Foxp3. These findings indicate that microbiota-driven remodeling of the bile acid pool can directly alter the Th17-Treg balance (Hang et al., 2019).

In a murine hepatocellular carcinoma model, gut microbiota-mediated bile acid transformation regulated the expression of C-X-C motif chemokine ligand 16 (CXCL16) in liver sinusoidal endothelial cells, thereby determining the accumulation of hepatic C-X-C motif chemokine receptor 6-positive (CXCR6^+^) natural killer T (NKT) cells and the interferon-gamma (IFN-γ) response, indicating that microbial metabolites can affect intrahepatic innate-like T-cell responses through the bile acid-CXCL16 axis (Ma et al., 2018). In addition, both preclinical NASH mouse models and patient liver tissue harbor auto-aggressive CXCR6^+^CD8^+^ T cells. Interleukin-15 (IL-15) first induces a FOXO1-low, CXCR6-high state in these cells, after which metabolic stimuli such as acetate and extracellular adenosine triphosphate (ATP) further trigger major histocompatibility complex class I (MHC-I)-independent hepatocyte killing through P2X purinoceptor 7 (P2X7). This suggests that the microbiota-associated metabolic milieu may amplify liver injury by lowering the pathogenic threshold of this T-cell subset (Du et al., 2021).

### 3.3 Epigenetic reprogramming

#### 3.3.1 DNA methylation

**FIGURE 5:**
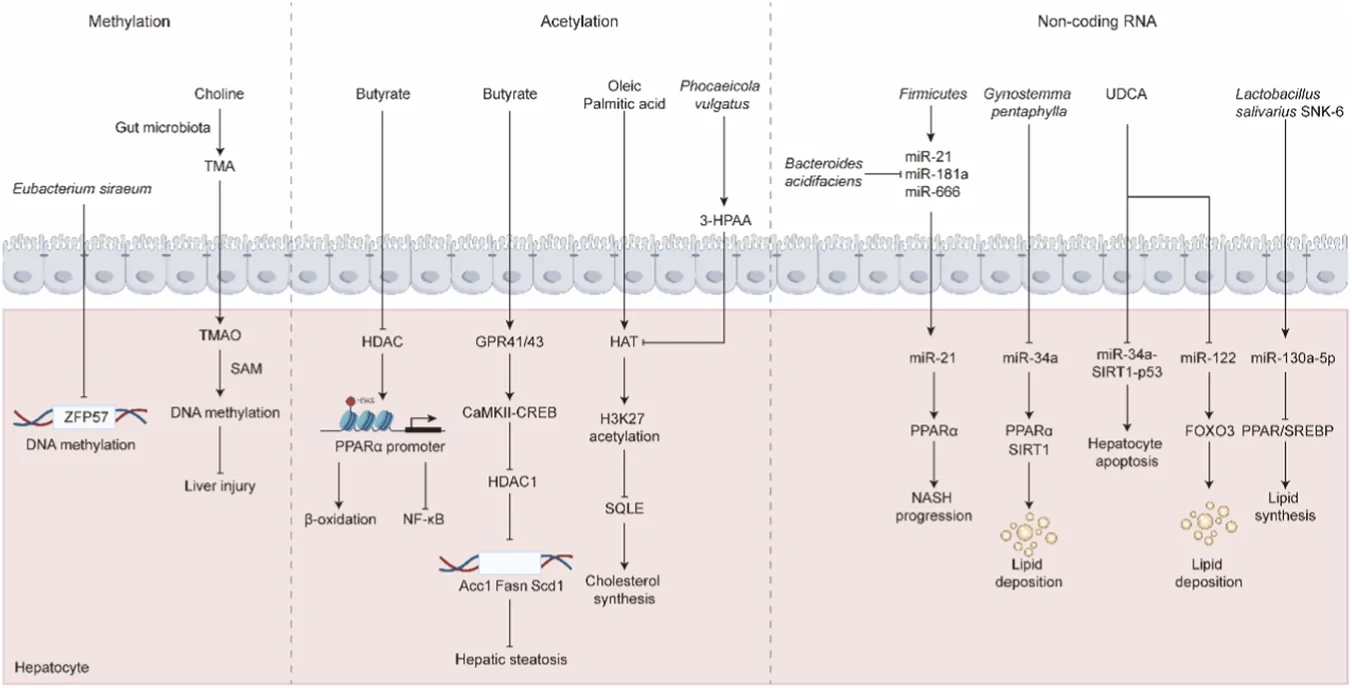
Gut microbiota-derived epigenetic regulation in MASLD. An inverse correlation was observed between the abundance of *Eubacterium siraeum* and ZFP57 methylation levels. The gut microbiota can convert choline into TMA, which is further transformed into TMAO, thereby affecting substrate availability for SAM-dependent DNA methylation. Supplementation with betaine, choline, or folate increases the hepatic SAM/SAH ratio and global DNA methylation levels, while concurrently alleviating liver injury. As an HDAC-inhibitory signal, sodium butyrate enhances H3K9 acetylation at the *Pparα* promoter, activates PPARα-mediated fatty acid β-oxidation, and suppresses the NF-κB inflammatory pathway. Butyrate can also activate hepatic GPR41/43-CaMKII-CREB signaling and inhibit HDAC1, thereby downregulating lipogenic genes such as *Acc1*, *Fasn*, and *Scd1* and attenuating hepatic steatosis. Oleic acid and palmitic acid enhance HAT activity and the expression of lipogenesis-related genes. 3-HPAA derived from *Phocaeicola vulgatus* inhibits hepatocellular HAT activity, reduces H3K27 acetylation, and downregulates *SQLE* transcription, thereby suppressing cholesterol synthesis. The relative abundance of Firmicutes is positively correlated with hepatic miR-21, miR-181a, and miR-666, whereas *Bacteroides acidifaciens* is negatively correlated with these miRNAs and hepatic triglyceride burden. miR-21 promotes NASH progression by suppressing PPARα. miR-34a targets PPARα and SIRT1, thereby inhibiting fatty acid oxidation and promoting hepatic lipid accumulation. *Gynostemma pentaphylla* intervention downregulates miR-34a and ameliorates hepatic steatosis. UDCA suppresses the miR-34a-SIRT1-p53 axis to alleviate hepatocyte apoptosis; it also reduces miR-122 levels, thereby releasing FOXO3 and improving lipid accumulation. *Lactobacillus salivarius* SNK-6 reverses the downregulation of hepatic miR-130a-5p and inhibits the PPAR/SREBP-related lipogenic pathway.

A study of allogeneic FMT in patients with NAFLD showed that, compared with autologous transplantation, FMT from healthy donors not only remodeled the recipients’ gut microbiota and choline-related metabolite profiles, but was also accompanied by changes in DNA methylation at hepatic loci such as TARS and ZFP57 (Figure 5). Among these, the abundance of Eubacterium siraeum was negatively correlated with methylation at a specific ZFP57 site, suggesting that microbiota remodeling may be transmitted to the level of hepatic DNA methylation (Stols-Gonçalves et al., 2023). In addition, a recent study of liver biopsy specimens from patients with MASLD found that hepatic bacterial DNA abundance was associated with total hepatic histone acetyltransferase (HAT) and histone deacetylase (HDAC) activity, whereas global hepatic 5-hydroxymethylcytosine (5-hmC) levels were negatively correlated with the abundance of Bacteroidetes and *Gammaproteobacteria*. A whole-genome methylation analysis of liver biopsy samples from a Japanese NAFLD cohort showed that cytosine-phosphate-guanine (CpG) modules associated with immune inflammation tended to be hypomethylated in advanced NAFLD, whereas modules related to mitochondrial function, lipid droplet homeostasis, and lipid metabolism tended to be hypermethylated, suggesting that aberrant methylation simultaneously links metabolic dysregulation and inflammatory amplification (Hotta et al., 2018). In liver tissue from patients with advanced NAFLD, hypermethylation of CpG99 within the PNPLA3 regulatory region was associated with reduced transcription, indicating that abnormal DNA methylation may impair lipid droplet remodeling and lipid turnover (Kitamoto et al., 2015). In liver tissue from patients with NASH, methylation of mitochondrial NADH dehydrogenase 6 (MT-ND6) was increased and accompanied by reduced expression and upregulation of DNA methyltransferase 1 (DNMT1). Ultrastructural abnormalities of mitochondria were also observed, suggesting that aberrant mitochondrial DNA (mtDNA) methylation may be associated with suppressed mitochondrial transcription and mitochondrial dysfunction. More recently, studies using liver biopsies from patients with MASLD and mouse models of MASH have shown that multiple complement cascade-related genes undergo differential methylation associated with disease severity, together with expression imbalance. These findings indicate that the impact of abnormal DNA methylation extends beyond metabolism-related genes to innate immune regulatory networks and may collectively contribute to the transition of MASLD toward inflammatory and progressive phenotypes (Pirola et al., 2013; Magdy et al., 2024).

In high-fat diet-induced NAFLD rats, supplementation with betaine, choline, or folic acid increased the hepatic SAM/S-adenosylhomocysteine (SAH) ratio and global DNA methylation levels, while simultaneously alleviating steatosis, dyslipidemia, and liver injury (Bakir et al., 2019). In high-fat diet-fed mice, hepatic betaine levels were reduced and accompanied by downregulation of cystathionine beta-synthase (CBS) and upregulation of betaine-homocysteine S-methyltransferase (BHMT), suggesting remodeling of hepatic one-carbon metabolism and methyl donor utilization, which may be associated with the development of hepatic lipid accumulation (Dahlhoff et al., 2013). In rats fed a high-fat, high-sucrose diet, methyl donor supplementation altered CpG methylation within the Fasn promoter and reduced hepatic triglyceride levels. Further methylation analyses also identified differential methylation at multiple genes involved in lipid metabolism, including Srebf2, Agpat3, and Esr1, suggesting that disruption of one-carbon metabolism may extend to the level of DNA methylation and thereby influence hepatic transcriptional programs governing lipid metabolism (Cordero et al., 2013a; Cordero et al., 2013b).

#### 3.3.2 Histone acetylation

Clinical and animal studies have shown that both patients with NAFLD and ovariectomized mice fed a high-fat diet exhibit gut microbial dysbiosis and reduced butyrate levels, whereas FMT or butyrate supplementation alleviates hepatic steatosis (Liu et al., 2022). Studies in germ-free mice have shown that microbial colonization can remodel histone modification profiles in the proximal colon, liver, and white adipose tissue, particularly by affecting multiple histone H4 acetylation sites (Krautkramer et al., 2016). However, in conventionally housed adult mice, antibiotic depletion of the gut microbiota markedly reduced luminal and portal SCFA levels but did not significantly alter global histone acetylation in the liver, suggesting that the influence of microbial metabolites on hepatic acetylation may not manifest as synchronized global fluctuations. In MASLD, therefore, tissue-specific and locus-specific acetylation remodeling may be of greater relevance (Saiman et al., 2021). In diet-induced obese mice, intestinal epithelial-specific deletion of HDAC3 improves obesity-related metabolic abnormalities and is accompanied by reduced serum triglycerides and hepatic fat accumulation. By contrast, commensal bacteria can also generate inositol trisphosphate (InsP3) through phytate metabolism, thereby activating intestinal epithelial HDAC3 and counteracting the inhibitory effect of butyrate on HDAC activity, indicating that the gut microbiota does not suppress deacetylation in a unidirectional manner but dynamically modulates HDAC activity through distinct metabolites (Wu et al., 2020). In addition, study in high-fat, high-cholesterol (HFHC)-induced MASLD mice and hepatocytes have shown that 3-hydroxyphenylacetic acid (3-HPAA) derived from *Phocaeicola vulgatus* suppresses hepatocellular HAT activity, reduces histone H3 lysine 27 (H3K27) acetylation, and downregulates squalene epoxidase (SQLE) transcription, thereby attenuating cholesterol synthesis-associated lipid deposition (Jin et al., 2024).

#### 3.3.3 Non-coding RNA regulation

In high-fat diet-fed mice, the relative abundance of Firmicutes is positively correlated with hepatic microRNA-21 (miR-21), miR-181a, and miR-666, whereas *Bacteroides acidifaciens* is negatively correlated with these microRNAs and with hepatic triglyceride burden (Blasco-Baque et al., 2017). In human NASH liver tissue and experimental models, miR-21 is upregulated together with downregulation of PPARα. Further inhibition of miR-21 restores PPARα expression and attenuates liver injury, inflammation, and fibrosis, and this effect is lost in the absence of PPARα, indicating that miR-21 promotes NASH progression by suppressing PPARα (Loyer et al., 2016). In high-fat diet-fed mice treated with *Gynostemma pentaphylla*, improvement in hepatic steatosis is accompanied by remodeling of the gut microbiota and downregulation of hepatic miR-34a (Jia et al., 2018). In free fatty acid (FFA)-induced steatotic hepatocytes and liver tissue from high-fat diet-fed mice, miR-34a is upregulated and promotes hepatic lipid accumulation by suppressing fatty acid oxidation through direct targeting of PPARα and sirtuin 1 (SIRT1) (Ding et al., 2015). At the clinical level, plasma miR-582-3p is elevated in patients with NASH and is associated with differences in gut microbial distribution. Furthermore, in transforming growth factor beta-1 (TGF-β1)-treated LX-2 cells, miR-582-3p directly targets transmembrane BAX inhibitor motif-containing 1 (TMBIM1), promoting hepatic stellate cell activation while inhibiting apoptosis, which suggests that microbiota-associated microRNA abnormalities may contribute to NASH progression by enhancing the profibrotic phenotype of hepatic stellate cells (Huang et al., 2024). In another study, Tmbim1 was shown to suppress steatosis and inflammation in mouse and monkey models of NAFLD/NASH by promoting lysosomal degradation of TLR4, indicating that TMBIM1 itself exerts a clear hepatoprotective effect. Therefore, suppression of TMBIM1 by miR-582-3p may not only be associated with hepatic stellate cell activation, but may also further weaken hepatic defense against inflammatory and metabolic stress (Huang et al., 2024; Zhao et al., 2017). In addition, the small intestinal microbiota can directly determine host adaptation to dietary lipid digestion and absorption, and jejunal microbiota derived from high-fat diet-fed donors can transfer the high-fat absorption phenotype to recipient germ-free mice, suggesting that the gut microbiota can shape the intensity of lipid influx at the intestinal epithelial level (Martinez-Guryn et al., 2018). Further mechanistic studies have shown that, in addition to microRNAs, the gut microbiota can also participate in this process through long non-coding RNA (lncRNA). In mouse small intestinal epithelial cells, the microbiota suppresses small nucleolar RNA host gene 9 (Snhg9), thereby enhancing peroxisome proliferator-activated receptor gamma (PPARγ) signaling and upregulating lipid metabolism-related genes such as Cd36, Fabp4, and Scd1, ultimately promoting lipid absorption and storage (Wang et al., 2023).

Gut microbial metabolites can likewise mediate gut-liver axis reprogramming through non-coding RNA. In high-fat diet-induced NAFLD mice and FFA-stimulated alpha mouse liver 12 (AML12) cells, sodium butyrate and a gut microbiota-derived metabolite preparation both upregulate miR-150 while suppressing C-X-C chemokine receptor type 4 (CXCR4). Given that the chemokine (C-X-C motif) ligand 12/CXCR4 (CXCL12/CXCR4) axis mediates recruitment of CD4^+^ T cells to the liver in NASH mice and patients, these findings suggest that microbiota-derived metabolites may influence NAFLD-related metabolic inflammation through the miR-150/CXCR4 axis (Zhang et al., 2021; Boujedidi et al., 2015). Another microbiota-associated metabolite, ursodeoxycholic acid (UDCA), has also been shown to exert non-coding RNA-related regulatory effects in NAFLD. Human NAFLD liver tissue indicates activation of the miR-34a-SIRT1-p53 axis with increasing disease severity, whereas this axis is suppressed in the livers of rats and primary hepatocytes treated with UDCA, suggesting that UDCA may exert protective effects by alleviating hepatocyte apoptosis-related signaling (Castro et al., 2013). In overweight subjects with elevated transaminases, repeated administration of UDCA also reduces circulating microRNA-122 (miR-122) levels (Yoon et al., 2021). Meanwhile, in high-fat diet-fed mice and palmitic acid-treated L02 cells, inhibition of miR-122-5p improves lipid accumulation and alleviates inflammation and oxidative injury by releasing forkhead box O3 (FOXO3), suggesting that alterations in miR-122 may not be merely epiphenomenal but may be mechanistically involved in MASLD-related injury phenotypes (Hu et al., 2022). In addition, in an NAFLD model of laying hens, *Lactobacillus salivarius* SNK-6 reverses the downregulation of hepatic miR-130a-5p, targets membrane bound O-acyltransferase domain containing 2 (MBOAT2), and suppresses PPAR/sterol regulatory element-binding protein (SREBP)-related lipogenic pathways. The enrichment of SCFAs and bile acids in its culture supernatant further suggests that the gut microbiota may link gut-derived metabolic signals to hepatic lipid synthesis through the miR-130a-5p/MBOAT2 axis (Zhu et al., 2022).

## 4 Microbiota-targeted therapeutic strategies for MASLD

### 4.1 Fecal microbiota transplantation

In high-fat diet-induced steatohepatitis mice, 8 weeks of total FMT after disease establishment increased Christensenellaceae and *Lactobacillus*, accompanied by elevated cecal butyrate, restoration of intestinal epithelial ZO-1, and attenuation of endotoxemia. At the same time, hepatic IFN-γ and IL-17 were reduced, whereas Foxp3, IL-4, and IL-22 were increased, ultimately leading to decreased lipid deposition and lower NAFLD activity score (NAS). These findings suggest that in this model, FMT simultaneously improves intestinal barrier dysfunction and intrahepatic immune imbalance (Zhou et al., 2017). In high-fat diet-fed mice, multidonor FMT showed stronger effects than single-donor FMT. In addition to increasing beneficial taxa such as *Blautia* and *Akkermansia*, it restored barrier-related proteins including claudin-1, occludin, and E-cadherin, reduced serum LPS, and downregulated hepatic IL-1β, IL-6, TNF-α, and IFN-γ, suggesting that its major benefit lies not only in improving steatosis but also in reshaping the gut-liver axis through barrier repair and attenuation of gut-derived inflammatory signaling (Shou et al., 2023).

In experimental NASH mice induced by a high-fat, high-fructose, high-cholesterol (HFHCF) diet, allogeneic FMT reduced inflammation- and fibrosis-related mediators, upregulated hepatic nuclear factor erythroid 2-related factor 2 (NRF2), and improved intestinal barrier function. The study further proposed that the remodeled intestinal environment after FMT may promote the production of beneficial metabolites, including 4-hydroxyphenylacetic acid, thereby contributing to hepatoprotection (Lee et al., 2023). Washed microbiota transplantation is a refined form of FMT in which fecal suspensions undergo automated microfiltration followed by repeated centrifugation and resuspension to enrich the microbial fraction (Zhang et al., 2020). This preparation enables the transplant dose to be quantified according to the amount of enriched microbiota rather than stool weight and has been associated with fewer transplantation-related adverse events than manually prepared FMT in inflammatory bowel disease cohorts (Zhang et al., 2020). In diet-induced MAFLD mice, washed microbiota transplantation upregulated C-X-C motif chemokine receptor 6 (CXCR6) expression on group 3 innate lymphoid cells (ILC3s) and promoted their migration to the liver through the CXCL16/CXCR6 axis. The liver-homing ILC3s subsequently alleviated hepatic steatosis and liver injury by secreting IL-22, suggesting that microbiota transplantation not only remodels the intestinal microecology but also reconstructs the hepatic immune microenvironment by regulating ILC3 liver homing (Zhong et al., 2024). Another 2025 study in high-fat diet-fed mice suggested that the beneficial effect of FMT on steatosis may also be related to restoration of hepatic group 1 innate lymphoid cells (ILC1s) and changes in tryptophan-related metabolic signaling. In that study, 6 weeks of FMT from healthy donors improved the trend toward hepatic steatosis and was accompanied by an increase in hepatic ILC1s and elevated serum indole-3-carbinol. Exogenous indole-3-carbinol also increased hepatic ILC1s and attenuated steatosis through aryl hydrocarbon receptor (AhR) activation, suggesting that FMT may act in part through an ILC1-related AhR metabolic pathway (Hou et al., 2025).

Another study in rats fed a high-fat, glucose-fructose diet (HFGFD) showed that although microbiota transplantation did not significantly improve NASH histology, it markedly alleviated portal hypertension by reducing intrahepatic vascular resistance and restoring Akt-endothelial nitric oxide synthase (Akt-eNOS)-related insulin signaling (García-Lezana et al., 2018). The efficacy of FMT is also influenced by donor microbial status and intervention stage. In high-fat diet-induced NAFLD mice, curative FMT from pectin-treated donor mice improved hepatic steatosis and was accompanied by increased SCFAs. In contrast, preventive FMT induced white adipose tissue browning but did not clearly improve hepatic steatosis (Houron et al., 2021). Similarly, in germ-free recipient models, microbiota from either high-fat diet non-responder donors or donors showing the strongest response to quercetin conferred protection against high-fat diet-induced NAFLD and blocked related gut-liver axis abnormalities in recipients. Likewise, transplantation of resveratrol-remodeled microbiota into high-fat diet-fed mice reduced body weight, alleviated steatosis and low-grade inflammation, and improved hepatic lipid metabolism (Porras et al., 2019; Wang et al., 2020).

Reverse evidence from human microbiota transplantation further indicates that donor microbiota itself may transfer MASLD susceptibility. Microbiota from patients with NAFL increased recipient susceptibility to high-fructose, high-fat diet-induced fatty liver, whereas NASH-associated microbiota containing high alcohol-producing *Klebsiella pneumoniae* could even directly induce NAFLD. Removal of this strain abolished the pathogenic effect. These findings indicate that FMT-based therapy for MASLD must rely on strict donor screening and exclusion of pathogenic microbial communities (Yuan et al., 2019; Burz et al., 2021).

Craven et al. compared allogeneic FMT with autologous FMT in 21 patients with NAFLD. The transplant material was prepared by mixing 2 g of donor or autologous stool with 125 mL of sterile saline and was delivered endoscopically into the distal duodenum. Allogeneic FMT did not significantly improve HOMA-IR or MRI-PDFF, but it reduced the lactulose-to-mannitol ratio in participants with increased small intestinal permeability at baseline, suggesting that its early effect may be more closely reflected by improvement in intestinal barrier function (Craven et al., 2020). Witjes et al. (2020) used allogeneic FMT from lean vegan donors once every 8 weeks for a total of three administrations, with paired liver biopsies obtained during 24 weeks of follow-up. Allogeneic FMT altered the gut microbiota and plasma metabolites and was accompanied by improvements in hepatic necroinflammatory scores and in the expression of liver genes related to inflammation and lipid metabolism, although clear therapeutic evidence for improvement in the overall NAFLD activity score was not established (Witjes et al., 2020). A multi-omics analysis based on the same cohort further showed that allogeneic FMT-induced changes in the gut microbiota and plasma metabolites were associated with alterations in hepatic DNA methylation, indicating that FMT may influence host responses through microbial metabolites and hepatic epigenetic regulation, although these findings remain mechanistic rather than confirmatory evidence of therapeutic efficacy (Stols-Gonçalves et al., 2023). Xue et al. enrolled 75 patients with NAFLD, and the FMT group received 200 mL of fresh bacterial suspension daily for three consecutive days. After 1 month, FibroScan controlled attenuation parameter values decreased in the FMT group but increased in the non-FMT group, while microbial diversity and Bacteroidetes-related indices shifted toward those of healthy controls, with more pronounced improvement in patients with lean NAFLD (Xue et al., 2022). Groenewegen et al. conducted a double-blind randomized controlled trial in 20 patients with MASLD, administering allogeneic or autologous FMT at weeks 0, 3, and 6, with follow-up to week 12. Allogeneic FMT did not significantly improve MRI-PDFF, glucose tolerance, or liver biochemical parameters, suggesting that although consecutive FMT can induce donor-associated microbial engraftment, its short-term clinical benefits remain inconsistent (Groenewegen et al., 2025). Overall, these clinical studies suggest that FMT may improve intestinal barrier function, reshape the gut microbiota, and modulate metabolic and hepatic molecular phenotypes in some patients with MASLD or NAFLD. However, its effects on core clinical outcomes, such as hepatic steatosis, insulin resistance, and liver biochemical indices, remain inconsistent. Interpretation of these findings is further complicated by substantial methodological heterogeneity among studies. Existing trials have differed in donor selection, sample preparation, administration route, and treatment frequency, employing single- or multidonor preparations, fresh bacterial suspensions, endoscopic or nasoduodenal delivery, and either single or repeated administrations (Craven et al., 2020; Witjes et al., 2020; Xue et al., 2022; Groenewegen et al., 2025). Such differences may alter the composition and viability of the transplanted microbiota and influence donor-strain engraftment. Notably, multidonor preparations do not necessarily eliminate donor-specific effects, as engraftment may still be dominated by strains from only a subset of donors (Wilson et al., 2021). Oxygen exposure and freeze–thaw processing may also reduce bacterial viability and modify the metabolic capacity of FMT preparations (Papanicolas et al., 2019). Thus, the variability in hepatic and metabolic outcomes may partly reflect differences in FMT preparation and microbial engraftment rather than biological efficacy alone. Future studies should therefore adopt more standardized protocols and incorporate strain-level engraftment analyses to improve comparability across trials (Ianiro et al., 2022).

### 4.2 Natural products targeting the gut–liver axis

Among current traditional Chinese medicine-based interventions targeting the gut-liver axis in MASLD, WLT, a formulation centered on white peony in combination with licorice, has been shown in HFHS-induced C57BL/6J mice to exert a partial anti-NAFLD effect through remodeling of the gut microbiota (Table 1). Its effects included attenuation of hepatic steatosis, improvement of intestinal integrity, and downregulation of hepatic genes involved in Toll-like receptor-mediated inflammatory pathways (Chen et al., 2021). Earlier work on total glucosides of paeony in Sprague-Dawley rats fed a high-fat, high-sugar, high-salt diet showed that this intervention improved insulin resistance, corrected dyslipidemia and liver dysfunction, and attenuated oxidative stress, suggesting a protective effect against insulin resistance-related fatty liver (Zheng et al., 2008).

**TABLE 1:** Traditional Chinese Medicine-based interventions targeting the gut–liver axis in MASLD.

Intervention	Model	Level (preclinical)	Effects	References
WLT (white peony–licorice formulation)	HFHS-induced NAFLD in C57BL/6J mice	Animal study	Attenuated hepatic steatosis, improved glucose intolerance and systemic inflammation, restored intestinal integrity, and downregulated hepatic genes involved in Toll-like receptor-mediated inflammatory pathways; effects were at least partly associated with gut microbiota remodeling.	Chen et al. (2021)
Total glucosides of paeony (TGP)	High-fat, high-sugar, high-salt diet-induced fatty liver in Sprague-Dawley rats	Animal study	Improved hyperinsulinemia and insulin resistance, corrected dyslipidemia, reduced liver injury markers, and alleviated oxidative stress, indicating protection against insulin resistance-related fatty liver.	Zheng et al. (2008)
Jiangzhi Granules (JZG)	High-fat diet-induced NAFLD in mice	Animal study	Improved hepatic steatosis, liver function, and insulin resistance; dose-dependently corrected gut microbial dysbiosis, with reduced Desulfovibrionaceae and increased S24_7 and Lachnospiraceae in the high-dose group; also suppressed microbiota-related LPS biosynthesis and sulfur metabolism signals and modulated hepatic fatty acid oxidation- and inflammation-related pathways.	Wang et al. (2021)
Berberine	High-fat diet-fed BALB/c mouse NASH model	Animal study	Restored *Bifidobacterium* abundance and the Bacteroidetes/Firmicutes ratio; reduced body weight, blood glucose, serum lipids, insulin resistance, aminotransferases, and NAFLD activity score; downregulated hepatic CD14, IL-1, IL-6, and TNF-α.	Cao et al. (2016)
Berberine	High-fat diet-induced obese rats	Animal study	Reduced hepatic steatosis and metabolic abnormalities; reversed HFD-induced gut dysbiosis, including reduced *Escherichia coli* and lower plasma LPS; inhibited hepatic LPS/TLR4/TNF-α signaling and restored insulin receptor and IRS-1 expression.	Liu et al. (2018)
Berberrubine	High-fat diet-fed mice and oleic acid-treated HepG2 cells	Animal + cell study	Attenuated hepatic lipid accumulation and insulin resistance; reduced lipid deposition in OA-treated HepG2 cells; regulated multiple glucose and lipid metabolism-related proteins; remodeled gut microbiota, with a microbial profile distinct from that induced by berberine.	Yang et al. (2022)
Berberine plus evodiamine	High-fat diet-induced NAFLD in rats	Animal study	Improved hepatic steatosis and liver injury; increased beneficial gut bacteria and reduced potentially harmful taxa; inhibited ileal epithelial apoptosis, enhanced intestinal barrier-related proteins, and reduced LPS and inflammatory cytokines.	Dai et al. (2022)
2,3,5,4′-Tetrahydroxystilbene-2-O-β-D-glucoside (TSG)	Methionine-choline-deficient diet-induced NAFLD in C57BL/6J mice	Animal study	Reduced serum and hepatic lipid-related indices and liver injury markers; downregulated ASC and caspase-1; also improved gut microbial imbalance.	Han et al. (2018)
Alisol B	High-fat diet-induced MASLD in mice; AML-12 cells for mechanistic validation	Animal + cell study	Alleviated hepatic steatosis and liver injury; increased gut microbial diversity and *Akkermansia* abundance; corrected hepatic purine metabolic abnormalities, inhibited xanthine oxidase, and normalized metabolites such as hypoxanthine, allantoin, and inosine; XO overexpression weakened its lipid-lowering effect.	Yiyou et al. (2025)

In high-fat diet-induced NAFLD mice, Jiangzhi Granules dose-dependently corrected gut microbial dysbiosis. The high-dose group showed reduced Desulfovibrionaceae and increased S24_7 and Lachnospiraceae. Functional prediction of the microbiota and related assays further suggested that Jiangzhi Granules suppressed microbial outputs related to LPS and sulfur metabolism while additionally remodeling hepatic fatty acid oxidation- and inflammation-related signaling (Cao et al., 2016) Early studies of berberine in high-fat diet-fed BALB/c mice showed that it restored *Bifidobacterium* abundance and the Bacteroidetes/Firmicutes ratio, while downregulating hepatic CD14, IL-1, IL-6, and TNF-α, accompanied by improvement in NAS and metabolic parameters (Cao et al., 2016). In obese rats fed a high-fat diet, berberine was further shown to reverse the increase in *Escherichia coli* associated with elevated plasma LPS, inhibit hepatic LPS/TLR4/TNF-α signaling, and restore insulin receptor and insulin receptor substrate-1 (IRS-1) expression (Liu et al., 2018).

Berberrubine, a major metabolite of berberine, likewise attenuated lipid accumulation in high-fat diet-fed mice and oleic acid-treated HepG2 cells. This effect was accompanied by modulation of proteins involved in glucose and lipid metabolism as well as remodeling of the gut microbiota. Notably, the microbial profile enriched by berberrubine was not identical to that induced by berberine, suggesting that the parent compound and its gut microbiota-derived metabolites may act through different microbial configurations and host metabolic pathways (Yang et al., 2022). When combined with evodiamine, berberine reduced ileal epithelial apoptosis, decreased intestinal fatty acid-binding protein (I-FABP), upregulated occludin and ZO-1, and simultaneously lowered serum LPS and TNF-α, IL-1β, and IL-6 in high-fat diet-fed rats (Dai et al., 2022).

The *Polygonum multiflorum*-related component 2,3,5,4′-tetrahydroxystilbene-2-*O*-β-D-glucoside (TSG) downregulated apoptosis-associated speck-like protein containing a CARD (ASC) and caspase-1 in methionine-choline-deficient (MCD) diet-induced C57BL/6J mice, accompanied by improved microbial balance (Han et al., 2018) In high-fat diet-induced MASLD mice, Alisol B exerted protective effects through a dual mechanism involving restoration of gut microbial dysbiosis and correction of hepatic purine metabolic abnormalities. In addition to increasing the abundance of *Akkermansia* and related taxa, it inhibited xanthine oxidase (XO) and reversed abnormalities in hypoxanthine, allantoin, and inosine, whereas XO overexpression weakened its lipid-lowering effect (Yiyou et al., 2025).

## 5 Conclusions and future perspectives

Accumulating evidence indicates that MASLD is not merely a metabolic disease characterized by hepatic lipid deposition, but rather a complex disorder whose onset and progression are rooted in gut microbial dysbiosis, intestinal barrier disruption, and abnormal microbial metabolites. Alterations in the composition and function of the gut microbiota not only affect host metabolism through microbial metabolites, but can also further reshape hepatic transcriptional programs through DNA methylation, histone acetylation, and non-coding RNA-mediated regulation. In parallel, impaired intestinal barrier function and dysregulated immune responses cooperate with these processes to drive MASLD progression. Under certain conditions, FMT and natural products can improve microbial composition, repair the intestinal barrier, reduce endotoxin burden, and alleviate MASLD.

However, several critical issues remain unresolved. MASLD is markedly heterogeneous, and the dominant pathogenic mechanisms and actionable therapeutic targets may differ across disease stages, host metabolic backgrounds, and microbial configurations. In addition, the efficacy of FMT is influenced by factors such as donor selection and recipient microbial status. Although natural product-based interventions offer the advantage of multitarget regulation, their core mechanistic pathways and clinical reproducibility still require further validation. Future efforts to optimize donor selection, identify key metabolic pathways, and define the populations most likely to benefit from intervention may help shift MASLD management from empirical treatment toward more targeted precision therapy.
